# High prevalence of generalized ligamentous laxity in patellar dislocation with posterior weight-bearing lateral femoral condyle osteochondral fractures: an observational study and treatment outcomes

**DOI:** 10.1186/s13018-026-06748-w

**Published:** 2026-03-18

**Authors:** Zhixuan Nian, Sen Fang, Mingchun Li, Junwen Liang, Yijia Li, Ziting Wei, Liqiang Pan, Xudong Yang, Xiangdong Yun

**Affiliations:** 1 Department of Orthopaedics, Lanzhou University Second Hospital, No. 82 Cuiyingmen, Chengguan District, Lanzhou, Gansu China; 2 Orthopaedics Key Laboratory of Gansu Province, Lanzhou, China

**Keywords:** Patellar dislocation, Multiple ligament laxity, Lateral femoral condyle, Weight-bearing zone, Osteochondral fractures, Bioabsorbable implants

## Abstract

**Objective:**

To investigate the high incidence of multiple ligament laxity signs in a highly selected cohort of patients with patellar dislocation complicated by osteochondral fractures in the posterior weight-bearing zone of the femoral condyle, evaluate the therapeutic efficacy of absorbable cartilage pins, and further analyze the causes of such injuries.

**Methods:**

A retrospective review was performed using clinical data from 40 patients with patellar dislocation complicated by osteochondral fractures involving the posterior weight-bearing zone of the lateral femoral condyle who were admitted to the Second Hospital of Lanzhou University between January 2021 and August 2024. Demographic and clinical characteristics (age, sex, osteochondral fracture size, affected side, and follow-up duration) as well as baseline anatomical parameters, including patellar height (Caton–Deschamps index), tibial tubercle–trochlear groove (TT–TG) distance, and femoral anteversion angle, were recorded. Systemic ligamentous laxity was evaluated preoperatively using the Beighton score, and a Beighton score ≥ 4 was used to define multiple ligament laxity. Patellar tilt (PT) and patellar shift (PS) were measured on magnetic resonance imaging (MRI), whereas TT–TG was primarily assessed on computed tomography (CT), supplemented by MRI when necessary. All patients underwent osteochondral fragment reduction and fixation using absorbable cartilage pins in conjunction with medial patellofemoral ligament (MPFL) reconstruction.The prevalence of multiple ligament laxity in this cohort was described using a one-sample proportion test (exact binomial test) with literature-reported proportions as reference. The primary outcome was the Lysholm score at the final follow-up. A multivariable linear regression model was constructed with adjustment for baseline Lysholm score, age, sex, follow-up duration, and preoperative TT–TG distance. In addition, paired-sample t-tests were used to compare clinical outcomes (range of motion [ROM], Lysholm, International Knee Documentation Committee [IKDC], Tegner, and visual analog scale [VAS]) and radiological parameters (PT, PS, and TT–TG) between the preoperative assessment and the final follow-up. The clinical efficacy and postoperative complications associated with the combined absorbable cartilage pin fixation and MPFL reconstruction were subsequently evaluated.

**Results:**

All 40 patients completed follow-up (9–24 months; mean, 14.18 ± 4.9 months). All incisions healed primarily, and no predefined complications were observed. Follow-up imaging demonstrated fracture-site healing and stable fixation in all cases. Multiple ligament laxity (Beighton score ≥ 4) was present in 38 of 40 patients (95.0%); compared with literature-reported prevalence (approximately 20–30%), a one-sample exact binomial test showed a statistically higher proportion (*P* < 0.001), which should be interpreted as an enriched prevalence in this selected cohort. In multivariable linear regression, the Lysholm score at final follow-up was independently associated with baseline Lysholm score (β = 0.984, 95% CI 0.798–1.170; *P* < 0.001), whereas age, sex, follow-up duration, and preoperative TT–TG were not significant predictors (all *P* > 0.05). Significant improvements were observed from preoperative assessment to final follow-up in radiological parameters—PT (12.4 ± 2.5 vs. 5.7 ± 1.2), PS (9.7 ± 1.2 vs. 1.3 ± 0.5), and TT–TG (18.1 ± 2.3 vs. 14.6 ± 1.6)—and in clinical outcomes, including ROM (112.7 ± 5.3 vs. 128.4 ± 1.9), Lysholm (51.9 ± 2.6 vs. 82.7 ± 3.1), IKDC (43.1 ± 13.2 vs. 83.8 ± 6.8), Tegner (4.1 ± 1.2 vs. 5.9 ± 1.6), and VAS (7.23 ± 1.3 vs. 1.97 ± 0.62) (all *P* < 0.001).

**Conclusion:**

In the selective cohort of “patellar dislocation with concomitant osteochondral lesions in the posterior weight-bearing zone of the lateral femoral condyle” included in this study, multiple ligament laxity signs exhibited a high incidence rate. Although the current study design precludes exploration of the underlying mechanisms, the coexistence of ligament laxity with baseline anatomical features supports a plausible hypothesis: patients with multiple ligament laxity syndrome may exhibit altered patellofemoral kinematics and energy transfer pathways under dynamic loading. And early surgical intervention involving reduction and fixation with resorbable cartilage pins combined with medial patellofemoral ligament (MPFL) reconstruction may represent a reliable and feasible treatment strategy.

**Supplementary Information:**

The online version contains supplementary material available at 10.1186/s13018-026-06748-w.

## Introduction

Patellar dislocation occurs predominantly in adolescents, and acute traumatic patellar dislocation accounts for approximately 3% of knee joint disorders [1]. It is frequently accompanied by associated injuries, including cartilage damage of the medial patella or lateral femoral condyle, medial patellofemoral ligament injury, osteochondral fracture (OCF), and knee effusion [2]. If inadequately managed, patellar dislocation may result in chronic patellofemoral pain and, over time, progression to patellofemoral osteoarthritis [3]. Following patellar dislocation, approximately 30%–70% of patients sustain concomitant osteochondral injury, with osteochondral fragments predominantly originating from the medial articular surface of the patella and the anterolateral, non-weight-bearing region of the lateral femoral condyle; these locations are closely related to the collision trajectory associated with lateral patellar dislocation [3, 4]. Although most cartilage-bone injuries following patellar dislocation occur in the non-weight-bearing region anterior-lateral to the lateral femoral condyle, multiple studies indicate that damage may also involve the weight-bearing region posterior to the lateral femoral condyle [5–9]. This suggests that atypical contact mechanics may alter the injury pathway. Our team’s clinical observations also reveal that some patients develop osteochondral fractures (OCFs) in the weight-bearing region of the posterior lateral femoral condyle following patellar dislocation. This location falls outside the traditional dislocation trajectory, suggesting a different mechanism of injury compared to the conventional forward collision pattern seen in lateral patellar dislocations. Further observation revealed that most patients with patellar dislocation complicated by posterior lateral femoral condyle weight-bearing zone fractures exhibited multiple ligament laxity characteristics.

Multiple ligament laxity syndrome (Beighton score ≥ 4) [10] is regarded as a significant risk factor for patellar instability, as it markedly compromises dynamic stability of the patellofemoral joint and may lead to excessive lateral excursion and rotational displacement of the patella during weight-bearing knee flexion [11]. The occurrence of osteochondral fractures (OCFs) in the posterior weight-bearing zone of the lateral femoral condyle suggests that patellar dislocation in patients with multiple ligament laxity may involve more complex injury mechanisms. However, whether this specific injury location correlates with the degree of ligament laxity remains unclear, given the limited available evidence. Therefore, in patients with multiple ligament laxity syndrome—particularly those with patellar dislocation complicated by osteochondral fracture—the underlying injury mechanism should be prioritized to facilitate targeted management. Regarding fixation methods for patellar dislocation with osteochondral fragments, existing studies have demonstrated that fixation with absorbable cartilage pins is technically straightforward and is associated with favorable clinical outcomes [12, 13]. Accordingly, absorbable cartilage pins were used to fixate the avulsed osteochondral fragments during surgery.

Given the limited research on the incidence of multiple ligament laxity signs in patellar dislocation complicated by osteochondral fractures of the posterior weight-bearing zone of the lateral femoral condyle. This study aims to investigate the high incidence of multiple ligament laxity signs in a highly selected cohort of patients with patellar dislocation complicated by osteochondral fractures in the posterior weight-bearing zone of the femoral condyle. It further evaluates the therapeutic efficacy of absorbable cartilage pins and analyzes the underlying causes of such injuries.

## Materials and methods

### General information

We conducted a retrospective analysis of clinical data from 40 patients with patellar dislocation complicated by osteochondral fractures of the posterior weight-bearing zone of the lateral femoral condyle at Lanzhou University Second Hospital between January 2021 and August 2024:

Inclusion Criteria: (1) patients with patellar dislocation complicated by an osteochondral fracture involving the posterior weight-bearing zone of the lateral femoral condyle; (2) candidates for osteochondral fracture (OCF) fixation; and (3) availability of complete preoperative imaging documentation, including X-rays and MRI/CT scans.

Exclusion Criteria: (1) patellar dislocation without an osteochondral fracture involving the posterior weight-bearing zone of the lateral femoral condyle; (2) concomitant ligamentous injury of the knee joint; (3) prior knee surgery; and (4) other concomitant knee joint pathologies.

#### Definition and imaging-based identification of the posterior weight-bearing zone of the lateral femoral condyle

(1) Imaging localization and segmentation principles: On preoperative MRI (primarily sagittal sequences, with CT and/or coronal MRI used as needed), we selected the plane that most clearly depicted the lateral femoral condyle (LFC) articular surface and corresponded to the largest cross-sectional area of the lesion. The trochlear and condylar articular surfaces were first differentiated using anatomical landmarks, with the trochlear tip/condyle–trochlea transition serving as the boundary to exclude the typical anterior non–weight-bearing zone. We then measured the anteroposterior diameter of the femorotibial articular surface of the LFC (from the most anterior to the most posterior margin of the condylar articular surface) and divided this distance into three equal segments. The posterior one-third was defined as the posterior weight-bearing zone of the LFC. All imaging analyses were performed using the institutional PACS system. (2) Intraoperative verification: In all cases, arthroscopic exploration with the knee in maximal flexion allowed direct exposure of the lesion, confirming its location on the posterior weight-bearing articular surface of the LFC and corroborating the imaging-based localization. Both segmentation and lesion classification were independently performed by two senior associate chief physicians who were blinded to clinical outcomes.

#### Participants

Based on imaging findings and intraoperative exploration, a total of 40 patients were included: 15 males and 25 females, aged 13–25 years (mean age, 17.45 ± 3.5years). All patients sustained sports-related patellar dislocation complicated by osteochondral fractures in the posterior weight-bearing zone of the lateral femoral condyle. The OCF dimensions were (1.825 ± 0.211) × (1.656 ± 0.235) cm. Of these patients, 26 had left knee injuries and 14 had right knee injuries. 38 had Beighton scores of ≥ 4, whereas 2 had scores of < 4 (Table 1).

The Beighton score was completed at the time of initial patient enrollment/outpatient evaluation, both prior to surgical intervention. Scoring was performed independently by two senior orthopedic surgeons with extensive experience in knee sports medicine. Assessors received standardized training prior to scoring and remained unaware of patients’ imaging information throughout the evaluation process. Each item of the classic 9-point Beighton scale was assessed individually (bilateral little finger dorsiflexion, thumb-to-forearm contact, elbow hyperextension, knee hyperextension, and knee flexion with palm touching the floor). Elbow/knee hyperextension angles were determined using a standardized threshold (e.g., > 10°), with protractors used for measurement when necessary to minimize subjective error. A Beighton score ≥ 4 was defined as diagnostic for multiple ligament laxity [14].

**Table 1:** General data of the patients

Age (years)	Gender		Beighton Score (Example)		OCF size(cm)	Surgical Procedure	Affected limb (side)		Follow-up period (months)
	Male	Female	≥ 4	< 4			left	right	
17.45 ± 3.5	15	25	38	2	(1.825 ± 0.211) × (1.656 ± 0.235)	Fracture Reduction + MPFL Reconstruction	26	14	14.18 ± 4.9

#### Patient baseline anatomical parameters

(1) Patellar height: Measure patellar height using the Caton index [15] on lateral knee radiographs. A Caton index > 1.2 indicates a high-riding patella. (2) TT–TG: During CT measurement, the patient is positioned supine with the knee in near-extension for scanning. First, mark the line connecting the medial and lateral edges of the posterior femoral condyle as the reference line. Then mark the lowest point of the femoral trochlea and the midpoint of the tibial tubercle, measuring the perpendicular distance between these two lines. A TT-TG > 20 mm is defined as an abnormally increased TT–TG. (3) Femoral anteversion angle: Measured via CT of the lower limb with hip and knee joints in the same position. The angle formed between the line connecting the center of the femoral head and the center of the femoral neck and the tangent to the posterior edge of the femoral condyle is the femoral anteversion angle. Its normal range is 10°–25° [16] (Table 2).

**Table 2:** Patient baseline anatomical parameters

TT-TG (mm)	Patellar height (Caton index)		Femoral anteversion angle (°)	
18.14 ± 2.3	1.21 ± 0.29		26.1 ± 4.5	
	≥ 1.2	< 1.2	≥ 25	< 25
	30	10	35	5

#### Ethics

All patients underwent standardized preoperative evaluation and provided written informed consent. This study was conducted in accordance with the Declaration of Helsinki, and all methods complied with relevant guidelines and regulations. Ethical approval was obtained from the Medical Ethics Committee of the Second Hospital of Lanzhou University: No. 2025 A-1278 (Supplementary Material 1).

### Surgical procedures

**Fig. 1:**
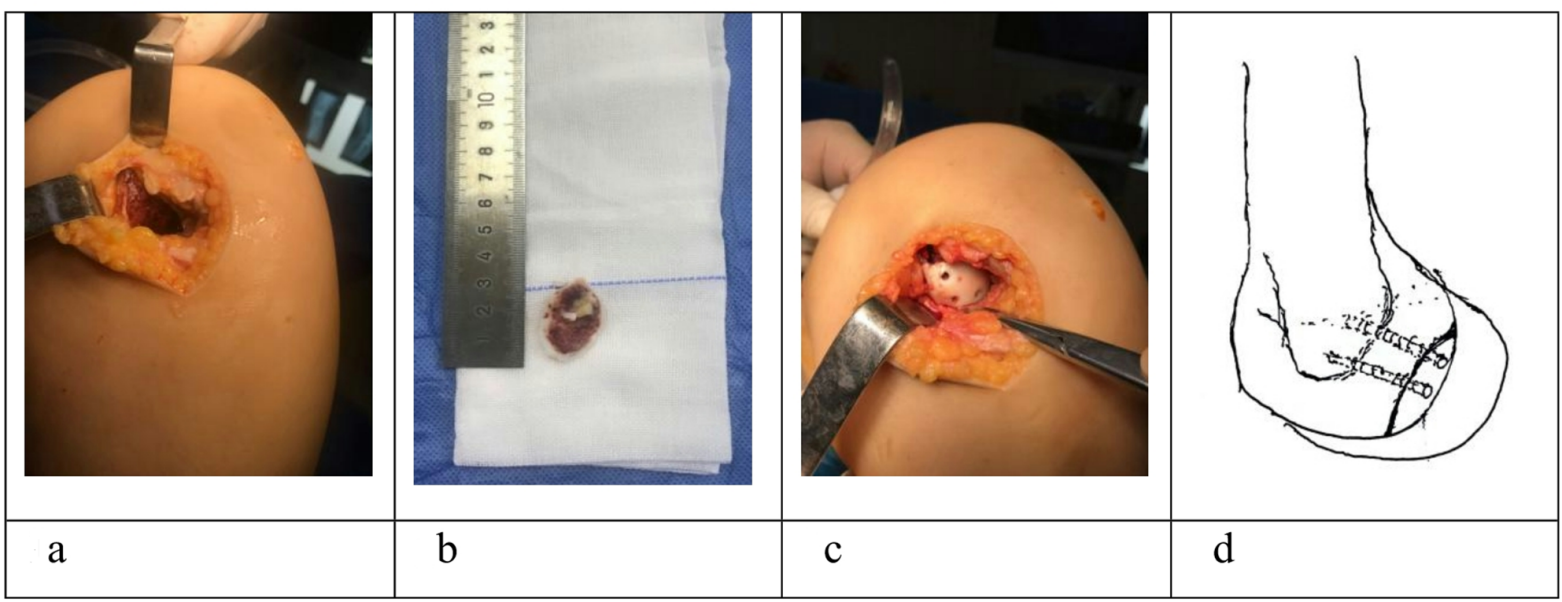
fracture of bone cartilage fracture in the posterior side of the femoral condyle,** a**: the bone cartilage defect;** b**: osteochondral fragments;** c**: bone cartilage fixation;** d**: the sketch map of absorbable cartilage screws fixation bone cartilage

All patients underwent general anesthesia in the supine position with the affected knee flexed at 90°, and standard aseptic preparation and draping were performed. Under anesthesia, the patellar lateral displacement test was reconfirmed to be positive. Knee arthroscopy was then performed, with two 0.8-cm portals established 0.5 cm lateral to the patellar tendon on each side to access the joint cavity, and the arthroscope was introduced through the lateral portal. The joint cavity, patellar articular surface, anterior and posterior cruciate ligaments, and menisci were examined sequentially to confirm a chondral fracture in the posterior weight-bearing zone of the lateral femoral condyle. The arthroscopic incision was subsequently extended to 3 cm both proximally and distally, and with the knee in maximal flexion, the posterior aspect of the lateral femoral condyle and the cartilage lesion site were exposed. For osteochondral fragments with a diameter > 1.5 cm, fixation was performed using absorbable cartilage screws. The semitendinosus tendon was harvested, residual muscle tissue was removed, and both ends were reinforced with continuous sutures for subsequent use. Using bone forceps, soft tissue and cartilage were cleared from the superomedial border of the patella, after which two 4.5-mm threaded anchors were inserted and the mid-substance of the ligament was secured to the anchors. Under C-arm fluoroscopy with a 30° axial projection, the patella was displaced medially and confirmed to slide readily into the trochlear groove, indicating laxity of the lateral patellofemoral retinaculum. An incision was then made through the skin and subcutaneous tissue between the medial femoral condyle and the adductor tubercle, and a 6-mm drill was used to create a 3-cm–deep bone tunnel. The graft tendon was pulled into the tunnel, the traction sutures were tensioned, and the knee joint was cycled repeatedly, after which the tendon was secured with a 6 × 25-mm compression screw. Finally, the surgical incision was closed in layers (Fig. 1).

### Postoperative management

The patient will wear a brace to immobilize the knee joint postoperatively, elevate the affected limb, and perform isometric contraction exercises for the quadriceps and hamstrings. The patient will be instructed to begin passive knee flexion exercises at 4 weeks postoperatively, with a range of motion (ROM) of 120°–130° at 6 weeks. Weight-bearing training will commence at 6 weeks, non-contact sports may be resumed at 3 months, and full return to previous activity levels is expected at 12 months.

### Follow-up and outcome assessment

#### Evaluation indicators

All patients underwent preoperative imaging studies: knee radiographs were used to assess patellar height (PH); knee MRI was used to evaluate patellar tilt (PT) and patellar shift (PS). Knee CT scans were used to measure the femoral anteversion angle and the bony TT-TG distance (combining CT and MRI findings when necessary). Follow-up evaluations were performed at 3, 6, and 12 months postoperatively and included reassessment of knee function together with radiographs, non-contrast computed tomography (CT), or magnetic resonance imaging (MRI).All radiographic measurements were performed within the hospital’s PACS system, adhering to predefined plane selection and measurement protocols to minimize measurement bias. In addition, the resorption of absorbable cartilage pins and osteochondral fragments was evaluated. Clinical outcomes were assessed using knee range of motion (ROM), the visual analogue scale (VAS) for pain, the Lysholm score, the International Knee Documentation Committee (IKDC) score, and the Tegner activity score. All data were independently measured by two senior orthopedic surgeons without knowledge of clinical outcomes.

#### Complication assessment

All patients underwent standardized clinical assessments at routine outpatient follow-ups and at the final follow-up, incorporating imaging data, with outcomes documented. We predefined the occurrence of any of the following events as “complication”: incision-related events (delayed healing, superficial/deep infection); internal fixation or fragment-related events (internal fixation instability/failure, fragment migration, nonunion); clinically significant postoperative stiffness (range of motion limitation); neurovascular injury; recurrent patellar instability/redislocation; and rehospitalization or reoperation related to this surgery. The absence of any of the above predefined events during the follow-up period is defined as “no complications.”

### Statistical analysis

Statistical analyses were performed using SPSS Statistics version 29.0 (IBM Corp.). Continuous variables were summarized as the mean ± standard deviation (SD). Paired-sample t-tests were used to compare preoperative and postoperative measurements. To describe the prevalence of multiple ligament laxity syndrome in this cohort, a one-sample proportion test was performed using reference proportions derived from previous reports. Given the limited sample size and the extreme observed proportion, an exact binomial test was employed to obtain robust P-value estimates.

To account for potential confounding in postoperative functional outcomes, a multivariable linear regression model was fitted for the primary outcome (Lysholm score at final follow-up), adjusting for the baseline Lysholm score, age, sex, follow-up duration, and preoperative TT–TG distance. Regression estimates are reported as β coefficients with 95% confidence intervals (CIs). A two-sided P value < 0.05 was considered statistically significant.

## Results

### Baseline characteristics

This study included 40 patients. Demographic characteristics and preoperative imaging features are summarized in Table 1 and Table 2. The patellar height was assessed using the Caton-Deschamps Index (CDI), with a mean value of 1.21 ± 0.29. CDI > 1.2 indicates a high-positioned patella [15]. Thirty patients (75.0%) met the criteria for increased patellar height, with no cases of low-positioned patella identified. The mean femoral anteversion angle was 26.1 ± 4.5°, with 35 patients (87.5%) exhibiting increased femoral anteversion (> 25°). Analysis of baseline data revealed a high proportion of patients in this cohort concurrently presenting with high patella and increased femoral anteversion, indicating a relatively concentrated distribution of these key anatomical factors within the study population.

### Analysis of the prevalence of multiple ligament laxity in this research cohort

In this highly selected cohort, multiple ligament laxity syndrome demonstrated a high prevalence, with 38 cases (38/40, 95%). Previous studies [17, 18] have reported that the prevalence of generalized ligament laxity (based on the Beighton score) in patients with patellar instability or dislocation is approximately 20%–30%. Analysis using a single-sample proportion test (exact binomial test, *P* < 0.001) confirmed that this high prevalence remains statistically significantly different. However, this comparison is descriptive and should be interpreted as an enriched prevalence within a selected cohort. It should be noted that comparisons with previous study proportions may be influenced by selection bias and patient population heterogeneity.

### Follow-up and comparison of postoperative functional scores

All patients were followed up for 9–24 months (mean 14.18 ± 4.9 months). All surgical incisions healed in the primary phase postoperatively. No complications such as infection, internal fixation instability, nonunion of fractures, muscle atrophy, or recurrent patellar dislocation were observed.Postoperative knee range of motion (ROM), Lysholm score, International Knee Documentation Committee (IKDC) score, Tegner activity score, and visual analogue scale (VAS) score improved significantly compared with preoperative values (*P* < 0.05) (Table  3).

**Table 3:** Comparison of all patients’ scores at preoperative and final follow-up visits ($\bar{x}$ ± s, scores)

	ROM(º)	Lysholm score	IKDC score	Tegner score	VAS score
Preoperative	112.7 ± 5.3	51.9 ± 2.6	43.1 ± 13.2	4.1 ± 1.2	7.23 ± 1.3
final follow-up	128.4 ± 1.9	82.7 ± 3.1	83.8 ± 6.8	5.9 ± 1.6	1.97 ± 0.62
*t*	− 16.2	− 48.15	− 24.690	− 7.1	13.63
*P*	< 0.001	< 0.001	< 0.001	< 0.001	< 0.001
*d*	2.56	10.7	3.91	1.12	2.16
95% CI	(13.74, 17.66)	(29.53, 32.07)	(37.37, 44.03)	(1.29, 2.31)	(− 6.04, − 4.48)

### Multivariable linear regression analysis of the Lysholm score at final follow-up

To further control for potential confounding factors, we performed a multivariate linear regression analysis on the Lysholm scores at the final follow-up. Using the final follow-up Lysholm score as the dependent variable, we constructed a multivariate linear regression model incorporating age, gender, follow-up duration, preoperative Lysholm score, and preoperative TT–TG distance (*n* = 40). Results demonstrated that preoperative Lysholm score was independently associated with final follow-up Lysholm score (β = 0.984, 95% CI 0.798–1.170; *P* < 0.001). Age (*P* = 0.900), gender (*P* = 0.922), follow-up duration (*P* = 0.609), and preoperative TT–TG distance (*P* = 0.585) showed no statistically significant correlation with Lysholm scores at final follow-up (Table 4).

**Table 4:** Multivariable linear regression for Lysholm score at final follow-up (n = 40).

Predictors	β	95% CI	*P* value
Preoperative Lysholm score	0.984	0.798 to 1.170	< 0.001
Age (years)	− 0.009	− 0.161 to 0.142	0.900
Sex (male vs female)	0.058	− 1.137 to 1.254	0.922
Follow-up duration (months)	0.037	− 0.108 to 0.181	0.609
Preoperative TT–TG distance (mm)	0.081	− 0.218 to 0.379	0.585

β: indicates adjusted regression coefficients. Female sex was used as the reference category. TT–TG: tibial tuberosity–trochlear groove distance. Follow-up duration is expressed in months.

### Postoperative imaging evaluation of absorbable cartilage screws

Postoperative imaging follow-up demonstrated significant reductions in PT, PS, and TT–TG values at final follow-up compared with preoperative measurements (*P* < 0.05). Moreover, imaging confirmed fracture-site healing at the posterior lateral femoral condyle in all patients, with no adverse events observed, including resorption of the fixed osteochondral fragments, recurrent patellar dislocation, or internal fixation instability; postoperative radiographic evaluation of the osteochondral fragments was satisfactory (Table 5).

**Table 5:** Comparison of preoperative and final follow-up imaging data ($\bar{x}$ ± s, Scores)

	PT(º)	PS (mm)	TT-TG (mm)
Preoperative	12.4 ± 2.5	9.7 ± 1.2	18.1 ± 2.3
Final follow-up	5.7 ± 1.2	1.3 ± 0.5	14.6 ± 1.6
*t*	− 7.1	− 38.7	− 8.24
*P*	< 0.001	< 0.001	< 0.001
*d*	1.12	6.12	1.30
95% CI	(− 8.61, − 4.79)	(− 8.84, − 7.96)	(− 4.41, − 2.67)

### Typical cases

**Fig. 2:**
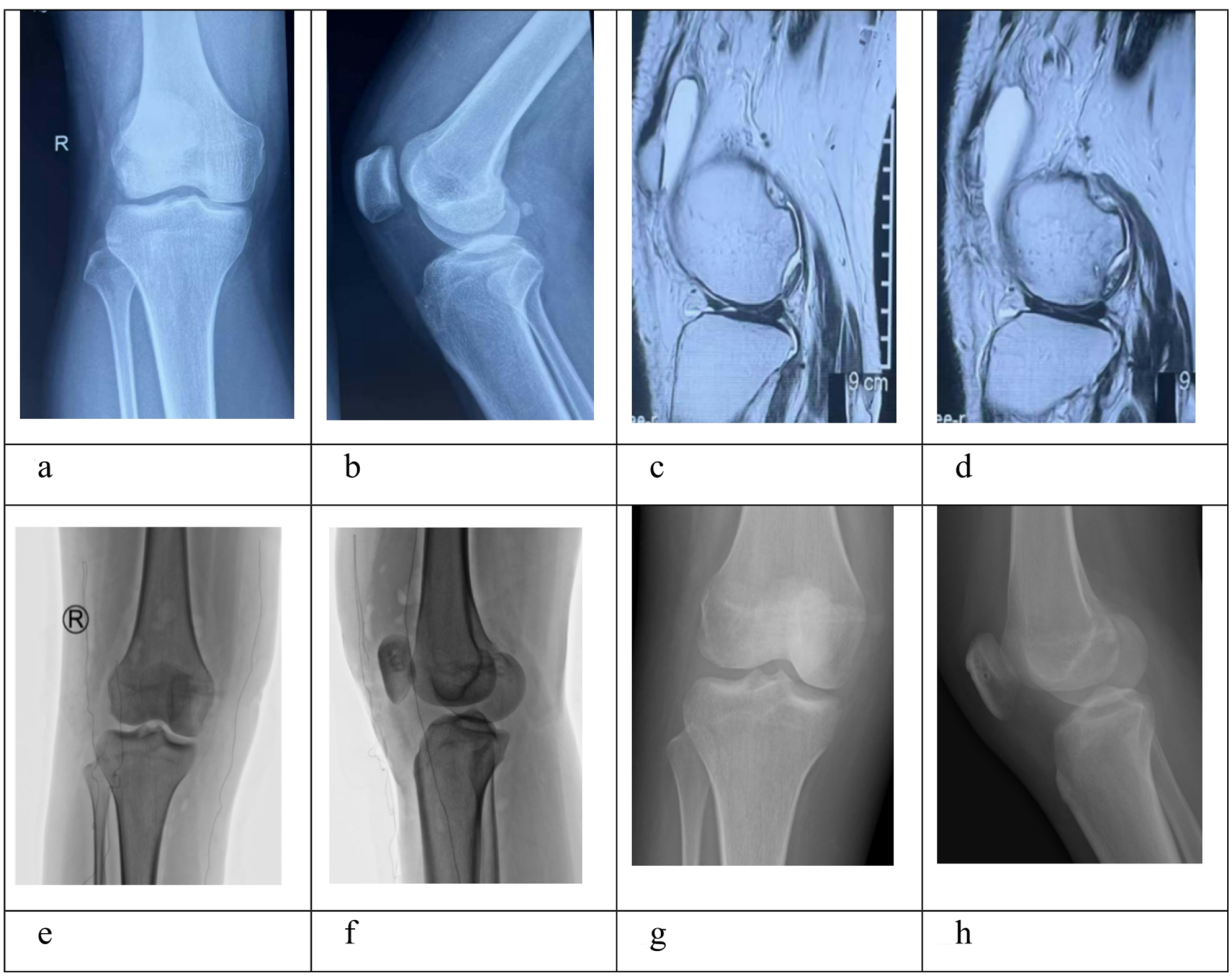
20-year-old female **a**,** b**: Preoperative X-ray showing posterior osteochondral fracture of the lateral femoral condyle; **c**,** d**: Preoperative MRI showing posterior osteochondral fracture of the lateral femoral condyle; **e**,** f**: Postoperative X-ray at 1 day showing OCF fixation achieved; **g**,** h**: Anteroposterior radiograph of the right knee at 12 months postoperatively demonstrates good reduction and union of the osteochondral lesion

A 20-year-old female patient with multiple ligament laxity (Beighton = 6) presented with right patellar dislocation and a posterior osteochondral fracture of the lateral femoral condyle. Preoperative radiographs and magnetic resonance imaging (MRI) demonstrated an osteochondral fracture involving the posterior weight-bearing zone of the lateral femoral condyle in the right knee. Open reduction of the osteochondral fracture and medial patellofemoral ligament (MPFL) reconstruction were performed. A postoperative radiograph obtained on postoperative day 1 showed stable fixation of the osteochondral fracture. At 12 months postoperatively, radiographs of the right knee demonstrated a well-reduced and healed osteochondral fracture, with a Lysholm score of 85 (Figure 2).

## Discussion

The principal finding of this study is that signs of markedly high prevalence of multiple ligament laxity were highly prevalent in this selected cohort of patients with patellar dislocation complicated by osteochondral injury in the posterior weight-bearing zone of the lateral femoral condyle. After the combined procedure (osteochondral fragment reduction/fixation and MPFL reconstruction), postoperative imaging demonstrated fracture-site healing and fragment stability in all patients at 9–24 months (mean, 14.18 ± 4.9 months) of follow-up. In terms of functional outcomes, postoperative scores improved significantly compared with preoperative values, with differences reaching statistical significance.Furthermore, to better control for potential confounding factors, we constructed a multivariate linear regression model using the Lysholm score at the last follow-up as the dependent variable. Results indicate that in the highly selected cohort included in this study, postoperative functional recovery was closely correlated with baseline functional status, while showing no statistically significant association with confounding factors such as age, gender, follow-up duration, or preoperative TT–TG.

### Potential mechanisms for cartilage fractures in the posterior weight-bearing zone of the lateral femoral condyle

Patellar dislocation complicated by osteochondral fracture is associated with multiple factors. Jiang et al. [19] identified male sex and an increased femoral anteversion angle as independent risk factors for patellar fractures complicated by OCF. Other studies have suggested that patients with TT–TG values > 15 mm, particularly > 20 mm, have the highest risk of recurrent instability [20]. This conclusion aligns closely with the preoperative imaging parameters observed in our study, further supporting the importance of the TT–TG value in predicting patellar stability. However, these risk factors—primarily related to osseous morphology and alignment—remain insufficient to fully explain the distinct injury pattern observed in our cohort, namely osteochondral fractures in the posterior weight-bearing zone of the lateral femoral condyle occurring in the setting of patellar dislocation. In the present cohort of 40 patients with patellar dislocation complicated by osteochondral fractures in the posterior weight-bearing zone of the lateral femoral condyle, the incidence of multiple ligament laxity syndrome was 95.0%, which was significantly higher than the approximately 20%–30% prevalence of generalized ligament laxity reported in previous studies of patients with patellar instability or dislocation [17, 18]. Both a one-sample proportion test and an exact binomial test, using P₀ = 0.25 as the expected value, indicated that the incidence of ligament laxity in this sample was significantly higher than previously reported estimates (*P* < 0.001). Together, these results suggest a high prevalence of multiple ligament laxity syndrome in this specific subtype of patellar dislocation complicated by osteochondral fractures in the posterior weight-bearing zone of the lateral femoral condyle. This finding indicates that ligament laxity may contribute to the injury mechanism, the risk of recurrent dislocation, and the development of osteochondral damage.

Previous biomechanical studies have suggested that lateral patellar dislocation most commonly occurs during knee extension with slight flexion (20°–30°). Callewier et al. [21] proposed that contraction of the vastus lateralis during this phase generates substantial lateral forces on the patella, which act on the trochlear articular surface and may predispose to trochlear–patellar fractures. However, this mechanism alone does not fully explain the occurrence of osteochondral fractures in the posterior weight-bearing zone of the lateral femoral condyle observed in certain patients.

In the present study, a striking clinical observation was that 95% of patients with patellar dislocation complicated by osteochondral fractures in the posterior weight-bearing zone of the lateral femoral condyle met the criteria for multiple ligament laxity syndrome, and all injuries occurred during sports activities. This clustering of ligamentous laxity suggests that factors beyond osseous morphology and alignment may contribute to this specific injury pattern.

Based on these observations, we propose that in patients with generalized ligamentous laxity, the knee joint may be more susceptible to excessive flexion and uncontrolled valgus and rotational forces during dynamic loading and rapid directional changes [22]. Following lateral patellar dislocation in mild flexion, diminished restraint from the medial retinaculum, joint capsule, and surrounding soft tissues may permit greater abnormal patellar translation along the lateral femoral condyle. As a result, impact forces may be transmitted posteriorly through the patellofemoral joint and concentrated on the posterior weight-bearing zone of the lateral femoral condyle [23], thereby increasing the risk of osteochondral fracture in this region. Collectively, these findings suggest that multiple ligament laxity syndrome may not only predispose patients to patellar dislocation but also exacerbate focal osteochondral damage by altering knee kinematics and energy transmission pathways. From a clinical perspective, routine assessment for generalized ligamentous laxity may therefore be valuable for risk stratification and for guiding preventive strategies, surgical planning, and rehabilitation protocols.

### Exposure and fixation of cartilage fractures in the posterior weight-bearing zone of the lateral femoral condyle

Fractures involving the posterior weight-bearing zone of the lateral femoral condyle can be visualized arthroscopically; however, substantial controversy remains regarding optimal exposure and fixation techniques [24]. Previous reports have indicated [25] that arthroscopic screw fixation from the proximal femur toward the joint cavity yields favorable outcomes for Hoffa fractures involving larger bony fragments. In contrast, for thinner chondral fractures, careful selection of appropriately sized screws is essential to minimize iatrogenic injury. Moreover, intra-articular arthroscopic fixation may fail to provide adequate visualization, thereby necessitating fixation via a tibial tunnel approach, which carries a recognized risk of iatrogenic damage to the tibial plateau cartilage [26].

In the present study, after arthroscopic confirmation of the fracture location, the knee was placed in maximal flexion, and a small incision was made by extending the lateral arthroscopic approach 3 cm proximally and distally to expose the lesion site. This maneuver enabled clear visualization of the chondral fracture in the posterior weight-bearing zone of the lateral femoral condyle. Two appropriately sized absorbable cartilage screws were subsequently used for fixation, providing more secure stabilization than fixation performed from the superolateral aspect of the lateral femoral condyle. In our cohort treated with the combined procedure, clinical and radiological outcomes were favorable, and this approach may streamline the surgical procedure while reducing operative time.

### Fixation options for cartilage fractures in the posterior weight-bearing zone of the lateral femoral condyle

The primary objectives of surgical fixation for osteochondral fractures are to restore articular surface congruency, provide adequate interfragmentary compression to facilitate healing, and ensure sufficient rotational stability to permit early postoperative range of motion. However, fixation strategies for chondral fractures remain highly variable, and the materials employed differ considerably among studies [12, 13, 27]. The absence of unified standards has introduced uncertainty regarding the selection of optimal fixation techniques and materials. Currently available fixation options include metallic screws [28], absorbable screws [29], and suture-based fixation techniques [30], each of which presents distinct biomechanical properties and potential limitations.

Previous studies have reported that absorbable fixation can achieve favorable clinical outcomes. Bradley et al. [31] demonstrated that absorbable fixation for adolescent knee osteochondritis was associated with high postoperative functional recovery, with 87.2% of patients achieving complete recovery. In contrast, although suture fixation for patellar fractures secondary to dislocation has shown initial efficacy, it has been associated with chondral nonunion during follow-up [32], and additional studies have suggested that PDS suture fixation may lead to postoperative fluid leakage through bone tunnels into the prepatellar bursa [33]. Based on these considerations, absorbable cartilage pins were selected in the present study to stabilize avulsed osteochondral fragments. This choice is further supported by the findings of Nudelman et al. [13], who reported excellent radiographic maintenance of fracture fragment position and no additional discomfort in patients with patellar dislocation complicated by osteochondral fractures treated with absorbable cartilage screw fixation at a mean follow-up of 12 months.

In the present study, fixation was achieved using two absorbable cartilage pins, and complete healing of the avulsed osteochondral fragment was observed during a minimum of 9 months of postoperative follow-up, accompanied by a significant improvement in the patient’s functional scores. Although the improvement in the patient’s subjective functional scores likely reflects the combined effects of articular surface restoration and stability reconstruction, rather than any single step alone, the compressive action of the absorbable cartilage pins ensured fracture site stability, thereby promoting the healing process. Furthermore, compared to certain metallic alternatives, absorbable cartilage pins may minimize iatrogenic damage to cartilage tissue during fixation. As the implants gradually resorb, they may promote cartilage regeneration and repair, thereby enhancing the quality of fracture healing. This approach offers a novel and effective treatment modality for patellar dislocation complicated by lateral femoral condyle cartilage fractures.

### Limitations of this study

This study also has the following limitations. (1) Selection bias is inherent to the study design. This cohort exclusively comprised a highly selected population with patellar dislocation accompanied by osteochondral fractures located in the posterior weight-bearing zone of the lateral femoral condyle. Accordingly, the observed high prevalence of multiple ligament laxity may not be generalizable to all patients with patellar dislocation or to osteochondral fractures at other classic locations. Therefore, these findings should be interpreted as an enriched co-occurrence of multiple ligament laxity within a specific injury phenotype, rather than as definitive evidence of association or causation. (2) The absence of a contemporaneous control group(e.g., patellar dislocation with osteochondral fractures at classic sites or patellar dislocation without osteochondral injury) precludes robust epidemiological comparisons and limits causal inference or strong associative conclusions regarding multiple ligament laxity and this specific injury pattern. (3) The statistical comparison framework has inherent constraints. The primary analyses were within-group preoperative to postoperative comparisons, which capture temporal changes but cannot exclude the influence of regression to the mean, natural disease course, or unmeasured confounding. Moreover, the one-sample proportion test comparing the prevalence of multiple ligament laxity in this cohort with literature-reported estimates may be affected by heterogeneity in study populations (e.g., age and sex distributions) and variability in assessment methods, as well as by the selection of the expected prevalence (P0). Although a multivariable linear regression model was introduced with Lysholm score at final follow-up as the outcome to provide limited adjustment, residual confounding remains likely given the retrospective design and sample size. (4) Imaging-based measurements are subject to potential variability. Radiological parameters may be influenced by differences in imaging modality, slice selection, knee positioning, and rotational alignment. Notably, changes in TT–TG observed in the absence of tibial tubercle–targeted correction may partially reflect measurement variability rather than true anatomical alteration. (5) All patients underwent concomitant osteochondral fracture fixation and MPFL reconstruction, which limits the ability to delineate the relative contribution of each procedure to the observed functional and radiographic improvements. Finally, the relatively short follow-up duration restricts assessment of long-term cartilage status and degenerative risk. Future studies with larger samples, multicenter designs, standardized imaging protocols, reliability testing, and appropriate control groups are warranted to validate these observations and elucidate potential mechanisms.

In summary, In the selective cohort of “patellar dislocation with concomitant osteochondral lesions in the posterior weight-bearing zone of the lateral femoral condyle” included in this study, multiple ligament laxity signs exhibited a high incidence rate. Although the current study design precludes exploration of the underlying mechanisms, the coexistence of ligament laxity with baseline anatomical features supports a plausible hypothesis: patients with multiple ligament laxity syndrome may exhibit altered patellofemoral kinematics and energy transfer pathways under dynamic loading. And early surgical intervention involving reduction and fixation with resorbable cartilage pins combined with medial patellofemoral ligament (MPFL) reconstruction may represent a reliable and feasible treatment strategy.

## Supplementary Information

Below is the link to the electronic supplementary material.

Supplementary Material 1

## Data Availability

The datasets used and/or analysed during the current study are available from the corresponding author on reasonable request.
