# Supplementary material for: High prevalence of generalized ligamentous laxity in patellar dislocation with posterior weight-bearing lateral femoral condyle osteochondral fractures: an observational study and treatment outcomes
Source: J Orthop Surg Res. 2026 Mar 18;21:319. doi: 10.1186/s13018-026-06748-w (PMC13244990; doi:10.1186/s13018-026-06748-w)
Supplement: Supplementary file 1 — Supplementary Material 1 [file 13018_2026_6748_MOESM1_ESM.pdf]

vThe Approval Document of the Institutional Medical Ethics Committee of the Second Hospital of Lanzhou University

Project Number: 2025A-1278

Project Title	A Study on the Correlation and Treatment Outcomes of Multiple Ligament Laxity Syndrome Combined with Patellar Dislocation and Posterior Weight-Bearing Zone Osteochondral Fractures of the Lateral Condyle of the Femur		
Sponsoring Institution	Lanzhou University, Lanzhou University Second Hospital		
Project Leader	Yun Xiangdong		
Department Responsible for Application	orthopedic surgery	Project Team Members	Nian Zhixuan, Fang Sen,Li Mingchun，Liang Junwen,, Li Yijia, Yang Xudong，Wei Ziting, Pan Liqiang
Project Type	☑ Article		
Review Category	☑ Initial Review ☐ Re-review	Review Format	☐ Meeting Review ☑ Expedited Review
Review Materials	Application Form, Research Proposal, Informed Consent Form, etc.		
Meeting Time	Nov.17 ,2025	Meeting Location	Floor Conference Room, Building , Inpatient Department, Lanzhou University Second Hospital
Chief Reviewer	☑Hongwen Zhu ☑Jing He ☑Xueliang Yan		
Review Conclusion	☑ Approved ☐ Approved after Necessary Corrections<br>☐ To be Discussed Again after Necessary Modifications<br>☐ Not Approved ☐ Terminated<br>☐ Suspended for Previously Approved Trials ☐ Recusal		
Frequency of Follow-up Review	☐ 3 months ☑ 6 months ☐ 12 months ☐ Not Applicable		
Review Opinion	The Committee of Medical Ethics Experts of the Second Hospital of Lanzhou University received the report of the project team .Conducted ethical review, questioning on the researcher's qualifications, project research programs, experimental informed consent, compensation, subject privacy protection and operational procedures. The participating ethics experts voted according to the review and the results are as follows:<br>Ethical review results:<br>Chairperson: 焦作义<br>Reviewing Entity (Stamped)<br>Review Date： Nov.17 ,2025<br>医学伦理委员会		
